# Exosomes as Conduits: Facilitating Hepatitis B Virus-Independent Hepatitis D Virus Transmission and Propagation in Hepatocytes

**DOI:** 10.3390/v16060825

**Published:** 2024-05-22

**Authors:** Marwa Khabir, Matthieu Blanchet, Léna Angelo, Hamza Loucif, Julien van Grevenynghe, Terence Ndonyi Bukong, Patrick Labonté

**Affiliations:** INRS–Centre Armand-Frappier Santé Biotechnologie, Laval, QC H7V 1B7, Canada; khabirmarwa@outlook.com (M.K.); matthieublanchet@hotmail.com (M.B.); lena.angelo@inrs.ca (L.A.); ha.loucif@gmail.com (H.L.); julien.vangrevenynghe@inrs.ca (J.v.G.); terencendonyi.bukong@inrs.ca (T.N.B.)

**Keywords:** hepatitis delta virus (HDV), exosomes, HBV-independent transmission

## Abstract

A number of research studies, including ours, have spotlighted exosomes as critical facilitators of viral dissemination. While hepatitis B virus (HBV) transmission through exosomes has been studied, the focus on its satellite virus, the hepatitis delta virus (HDV), has been unexplored in this context. HDV, although being a defective virus, can replicate its genome autonomously within hepatocytes, independently of HBV. Investigations on Huh7 cells revealed an intriguing phenomenon: the HDV proteins, S-HDAg and L-HDAg, are transmitted between cells without a complete viral structure. Detailed analysis further revealed that the expression of these proteins not only bolstered exosome secretion but also ensured their enrichment within these vesicles. Our experimental approach utilized transfection of various plasmids to examine the role of HDV RNA and proteins in the process. One salient finding was the differential propagation of the HDV proteins S-HDAg and L-HDAg, suggesting intricate molecular mechanisms behind their transmission. Notably, the purity of our exosome preparations was monitored using markers such as TSG101 and CD81. Importantly, these exosomes were found to carry both HDV RNA and proteins, highlighting their role in HDV dissemination. This novel study underscores the role of exosomes in mediating the transmission of HDV components between hepatocytes independent of HBV. These revelations about the exosomal pathway of HDV transmission provide a foundation for the development of innovative therapeutic strategies against HDV infections.

## 1. Introduction

Exosomes are nano-sized extracellular vesicles (50 nm to 150 nm) that are secreted by most cells [1,2,3]. They originate from the endosomal pathway and correspond to the intraluminal vesicles forming the multivesicular body (MVB) [4]. Originally, it was thought that exosomes were waste products released via the shedding of the plasma membrane [5]. Recently, several key roles of exosomes were demonstrated, such as cellular homeostasis [2], inflammation [6,7], and antigen presentation [8], ensuring, at least in part, cell-to-cell communication [9,10]. Indeed, it has been demonstrated that exosomes contain proteins, lipids but also a variety of nucleic acids (double-stranded DNA, mRNAs, microRNAs, and long noncoding RNAs) and that the contents of exosomes can be transferred to recipient cells and influence their functions [1,11]. Hence, exosomes mediate the transfer of bio-macromolecules, functional proteins, and nucleic acids between cells [4]. In addition to host components, exosomes can harbour pathogen-derived elements [12,13]. Interestingly, viruses have the ability to use the exosomal secretory pathways to promote virus spread and disease pathogenesis, like the human papillomavirus (HPV) [14], the human herpes virus 6 (HHV-6) [15], the herpes simplex virus 1 (HSV-1) [16], and the human immunodeficiency virus (HIV) [17,18]. Moreover, several publications have shown that other viruses, like the hepatitis C virus (HCV), hepatitis A virus (HAV) [19], hepatitis E virus (HEV) [20], and hepatitis B virus (HBV) [21], can be transmitted in a receptor-independent manner, via extracellular vesicles including exosomes [13,21,22]. Recently, the presence of hepatitis delta RNA in extracellular vesicles was reported [23], but their involvement in HDV transmission was not assessed.

Hepatitis delta is a severe form of chronic viral hepatitis [24], affecting between 12 and 72 million people worldwide [25,26,27]. It is caused by the hepatitis delta virus (HDV), a defective virus that needs the envelope protein from the HBV to form mature infectious virions [24]. Hepatitis B envelope proteins (HBsAg) occur as three isoforms: the small envelope protein (S-HBsAg), the medium (M-HBsAg), and the large (L-HBsAg). These isoforms are coded by either the S-domain only (S-HBsAg), the S-domain and an extension coded by the PreS2-domain (M-HBsAg), or the S, PreS2, and PreS1 domain (L-HBsAg) [28,29,30]. The HDV genome is a negative-strand RNA that contains one ORF, which encodes for the hepatitis delta antigen (HDAg). This protein is expressed as two isoforms: a small and a large HDAg. The 24 KDa small-HDAg (S-HDAg) is expressed early in the infection process and is important for the replication of the viral genome [31]. At a later stage of the infection, the edition of the amber stop codon of the S-HDAg to a tryptophane by the host double-stranded RNA adenosine deaminase (ADAR1) results in the addition of 19 amino acids and allows for the expression of the large isoform (27 KDa) of the protein (L-HDAg) [32]. L-HDAg is characterized by the presence of a nuclear export signal (NES) and of a CXXX motif within the 19 amino acid extension, allowing for its nuclear export and prenylation, respectively [33,34]. These signals are important for the formation and secretion of infectious viral particles [35]. Since they share a similar envelope, HBV and HDV use the same receptors to enter hepatocytes [36]. Indeed, they depend on the specific interaction of the PreS1 domain of L-HBsAg with the sodium taurocholate co-transporting polypeptide (NTCP), a multiple transmembrane transporter, expressed mainly in the liver [37]. HDV assembly relies on the specific interaction between the prenylated N-terminus of L-HDAg and the S-domain of HBsAg [35,38,39].

Recently, it was demonstrated that HDV could use other helper enveloped viruses, distinct from HBV, like HCV or Dengue virus, to egress from hepatocytes [40], suggesting that HDV could use unconventional cell transmission strategies and could be associated with different envelope glycoproteins. Since a recent study has confirmed that extracellular vesicles harbour HDV RNA [23], our current hypothesis is that HDV can also be transmitted by extracellular vesicles, including exosomes. We also postulated that this mechanism occurs in a receptor- and HBV-independent manner.

In this study, we showed that exosomes that were isolated from the supernatant of cells expressing HDV contain the two isoforms of HDAg, along with viral HDV RNA. We further observed that HDV components can be in vitro transmitted to non-infected hepatocytes in the absence of HBV or other helper viruses. We, therefore, suggest that HDV can be transmitted between cells through the exosomes.

## 2. Materials and Methods

### 2.1. Cell Culture, Reagents, and Antibodies

Huh7 cells were cultured in Dulbecco’s modified Eagle’s medium (DMEM, Gibco) and supplemented with 10% *v*/*v* fetal bovine serum (FBS), 100 U/mL penicillin, 100 μg/mL streptomycin, and 2 mM L-glutamine (Gibco). FBS Exosome-Depleted was purchased from Thermo Fisher Scientific. Exoquick-TC reagent (EXOTC10A-1) was purchased from System Biosciences. UNC7938 was a generous gift from Dr. Juliano [41] and was used as previously described [42,43,44].

Rabbit polyclonal anti-TSG101 was purchased from MyBioSource (MBS9201535), and mouse monoclonal anti-CD81 was purchased from Santa Cruz (sc-23962). Rabbit polyclonal anti-Calnexin (2679T) was bought from Cell Signaling. Anti-HDAg antibody-positive human serum was a gift from Dr. Sureau [38].

### 2.2. Plasmids and Transfection

HDV recombinant plasmid pSVLD3 was used for the replication of HDV RNA and production of HDV ribonucleoprotein (RNP). It contains three head-to-tail copies of full-length HDV cDNA [38]. The plasmids pCIHD24 and pCIHD27 were used for the expression of S-HDAg and L-HDAg, respectively, as previously described [38,45]. pCIneo and peGFP were purchased from Promega and Clontech, respectively. pcDNA3 mRuby2 (named in Section 3 pmRuby2) plasmid is from Addgene [46].

### 2.3. Exosome Isolation and Quantification

Supernatants from transfected cells cultured in DMEM supplemented with 5% exosome-depleted serum were collected and clarified. Then, supernatants were transferred into centrifugal Filter Units (Amicon Ultra centrifugal Filters, MilliporeSigma, Oakville, ON, Canada) and were centrifuged at 3500× *g* for 20 min. After serial filtrations of supernatants, the concentrated culture supernatants were mixed with the appropriate volume of Exoquick for exosome isolation according to the manufacturers’ specifications. The isolated exosomes were resuspended in 1X phosphate-buffered saline (PBS) and then either lysed with the appropriate lysis buffer, depending on the application, or conserved at −20 °C for further applications. Supernatants from transfected cells cultured in DMEM supplemented with 5% exosome-depleted serum were collected, clarified, and diluted in PBS to a final volume of 1 mL and analyzed with NANOSIGHT NS300. The purified exosomes were also quantified with NANOSIGHT NS300.

### 2.4. Flow Cytometry (FACS)

Cells were fixed with 4% paraformaldehyde (PFA) for 10 min. Then, they were permeabilized with 0.5% TritonX-100 and washed with FACS buffer with saponin (2% BSA, 0.1% sodium azide, 0.3% saponin). Cells were stained with anti-HDAg antibody-positive human serum at 1:1000 dilution for 1 h at 4 °C and incubated with the corresponding secondary Alexa fluor antibody for 1 h at 4 °C. Finally, samples were analyzed with Flow cytometry FACSDiva Version 6.2. The results were analyzed with FlowJo Version 10.6.2.

### 2.5. Quantitative Real-Time PCR

Intracellular RNA was extracted using an Aurum total RNA mini kit (BioRad, St-Laurent, QC, Canada), and reverse transcription was performed with iScript SELECT cDNA (BioRad). The quantification of HDV RNA was determined using SYBR Green real-time PCR. The primers used were as follows: forward HDV-F 5′-GGGATTTTCGTCCTCTATCTTC-3′, reverse HDV-R 5′-AGAGAAGAGATCCTCGAGCAG-3′.

### 2.6. Confocal Microscopy

Huh7 cells were transfected with the different plasmids as specified in the figure legend. Cells were grown on glass coverslips and fixed with 4% PFA. They were permeabilized with 0.2% TritonX-100 for 30 min and incubated with a blocking solution for 30 min (PBS, 3% bovine serum albumin, 10% FBS) at room temperature (RT). Coverslips were then incubated with anti-HDAg antibody-positive human serum at 1:1000 dilution for 1 h at RT, followed by incubation with the corresponding secondary Alexa fluor™ antibody for 1 h at RT. The nucleus was stained with DAPI for 15 min at RT and mounted on glass slides with ProLong Gold Antifade mountant (Invitrogen, Thermo Fisher, St-Laurent, QC, Canada). The coverslips were analyzed using confocal microscopy (Zeiss LSM 780, Dorval, QC, Canada).

### 2.7. Western Blotting

Cells or isolated exosomes were lysed in Pierce lysis buffer (25 mM Tris-HCl pH 7.4, 150 mM NaCl, 1% NP-40, 1 mM EDTA, 5% glycerol) and supplemented with protease inhibitor (Roche, Laval, PQ, Canada). The BCA protein assay kit (Pierce, Thermo Fisher, St-Laurent, Canada) was used to normalize the total protein content in the different lysates (cellular extract and exosome extract). Equivalent amounts of protein were subjected to sodium dodecyl sulfate-polyacrylamide gel electrophoresis (SDS-PAGE) and transferred to polyvinylidene fluoride (PVDF) membranes (BioRad). The membranes were blocked for 30 min at RT with PBS-5% milk and then incubated overnight at 4 °C with primary antibodies in PBS-0.1% BSA. Membranes were washed with 0.4% Tween 20 in PBS and were incubated for 1 h at RT with the corresponding secondary antibody conjugated to horseradish peroxidase in PBS-5% milk. Proteins’ bands were visualized with the Clarity western ECL (BioRad) using ChemiDoc XRS+ System (BioRad).

### 2.8. Statistical Analysis

The values were expressed as the mean ± the standard error of the mean of at least three independent experiments. The results were analyzed using either a two-tailed unpaired *t* test or one-way ANOVA followed by Tukey’s multiple comparisons tests. *p*-values below 0.05 were considered statistically significant (* *p* < 0.05; ** *p* < 0.01; *** *p* < 0.001; **** *p* < 0.0001). Statistical analyses were performed using GraphPad Prism 10.

## 3. Results

### 3.1. HBV-Independent Spreading of S-HDAg and L-HDAg In Vitro

**Figure 1:**
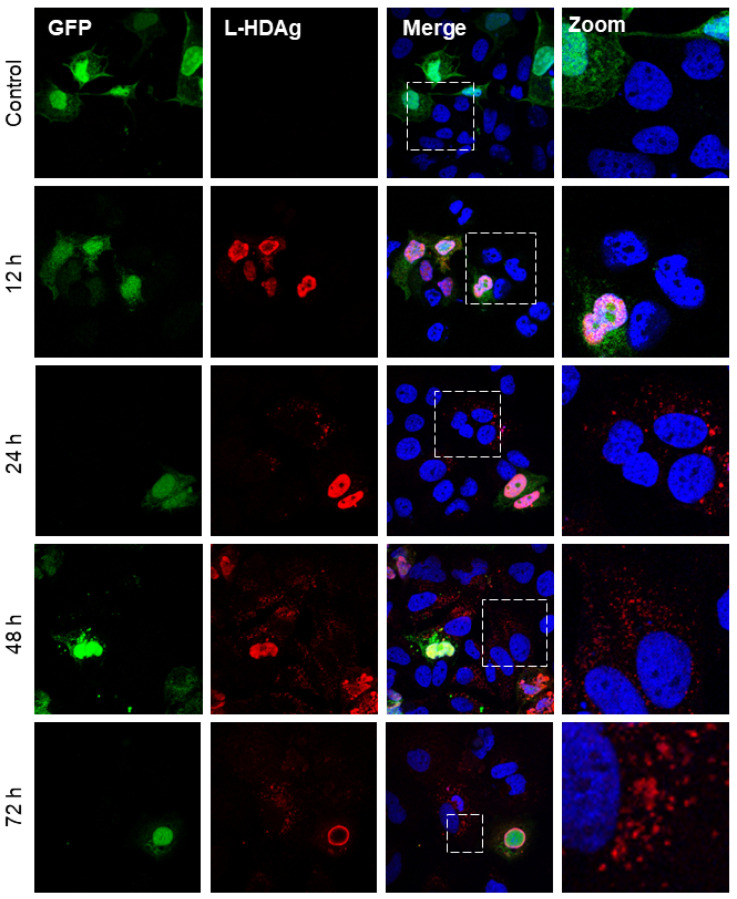
HBV-independent spreading of L-HDAg in vitro. Huh7 cells were co-transfected with peGFP plasmid and pCIHD27 coding for GFP protein and L-HDAg, respectively. At indicated time points, cells were fixed and then stained with anti-HDAg antibodies from human serum (red) and with DAPI (blue). The results were analyzed by confocal microscopy, and a representative example is shown for each time point. As a negative control, we co-transfected the cells with peGFP and pCIneo plasmids. L-HDAg staining at 12 h, 24 h, 48 h, and 72 h.

**Figure 2:**
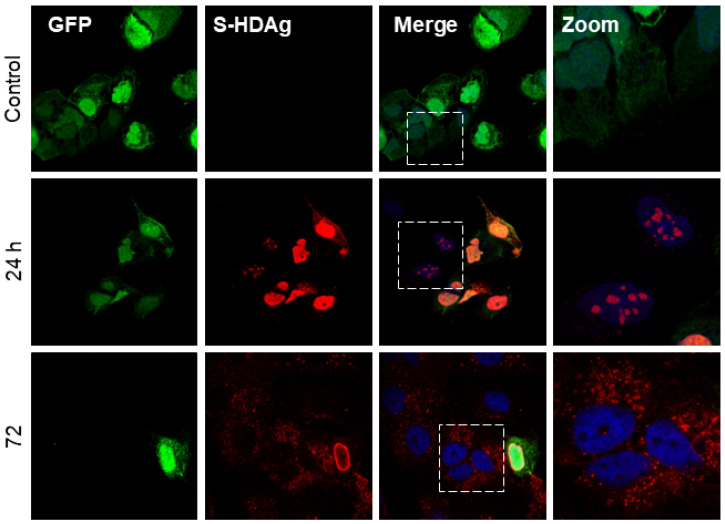
HBV-independent spreading of S-HDAg in vitro. Huh7 cells were co-transfected with peGFP plasmid and pCIHD24 coding for GFP protein and S-HDAg, respectively. At indicated time points, cells were fixed and then stained with anti-HDAg antibodies from human serum (red) and with DAPI (blue). The results were analyzed using confocal microscopy, and a representative example is shown for each time point. As a negative control, we co-transfected the cells with peGFP and pCIneo plasmids. S-HDAg staining at 24 h and 72 h.

Although HDV is a defective virus relying on the HBV envelope protein to form infectious particles, and it can replicate its genome independently [47]. The replication of HDV RNA results in the production of the two isoforms of HDAg. Interestingly, after the co-transfection of Huh7 cells with plasmid coding for GFP and L-HDAg (pCIHD27), we observed a significant proportion of Huh7 cells positive for L-HDAg but not GFP (Figure 1). This indicates that transfected cells were able to transfer L-HDAg independently from GFP to naïve cells independently of a full viral structure. The same observation was made in Huh7 cells co-transfected with plasmids coding the GFP and the S-HDAg (pCIHD24) (Figure 2). Moreover, the propagation of S-HDAg and L-HDAg to the surrounding cells increases over time (Figure 1 and Figure 2). As shown in Figure 1, L-HDAg can be detected in surrounding cells at 24 h post co-transfection and the propagation of L-HDAg is intensified at 48 h and 72 h. Consequently, for Figure 2, we only evaluated the propagation of S-HDAg at 24 h and 72 h. Of note, the assessed propagation of the HDAg was not affected by any unspecific background since we did not detect any staining against HDAg in cells co-transfected with peGFP and pCIneo (negative control).

**Figure 3:**
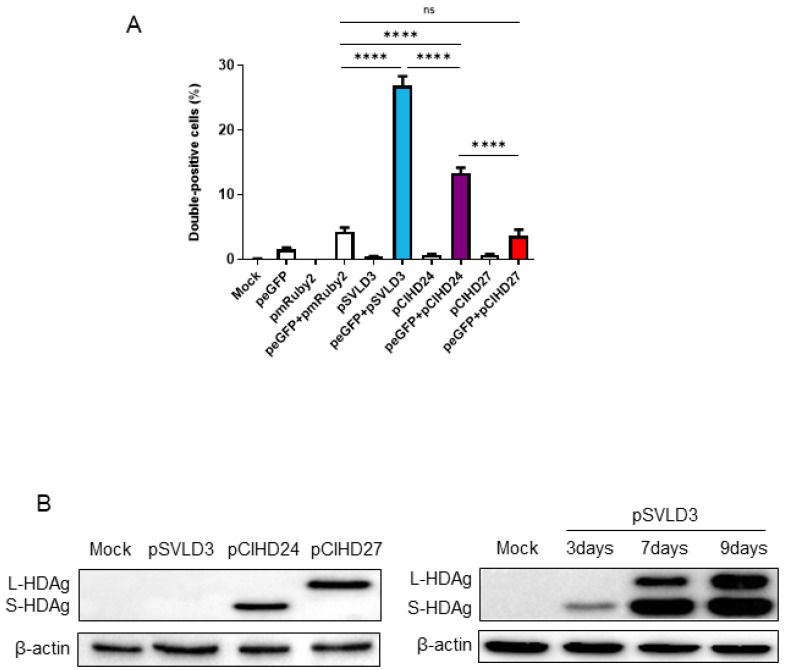
Quantification of double-positive cells for HDAg and GFP. (**A**) Huh7 cells were transfected with pSVLD3, pCIHD24, pCIHD27, or an empty plasmid, which was used as a negative control. Then, the expression levels of HDAg were evaluated using western blot. (**B**) Huh7 cells were transfected with pmRuby2, pSVLD3, pCIHD24, or pCIHD27 separately. These cells were then mixed with GFP-expressing cells and incubated for 72 h. After fixing, the cells were stained with anti-HDAg antibodies from human serum and analyzed using FACS. Untransfected mock cells stained with an anti-HDAg antibody from human serum were used as a control for settings. The percentages of double-positive cells (GFP+ and HDAg+) for the cells transfected by pSVLD3, pCIHD24, and pCIHD27 were 27%, 13%, and 4%, respectively. The experiment was repeated at least three times. ns, not significant; **** *p* < 0.0001.

**Figure 4:**
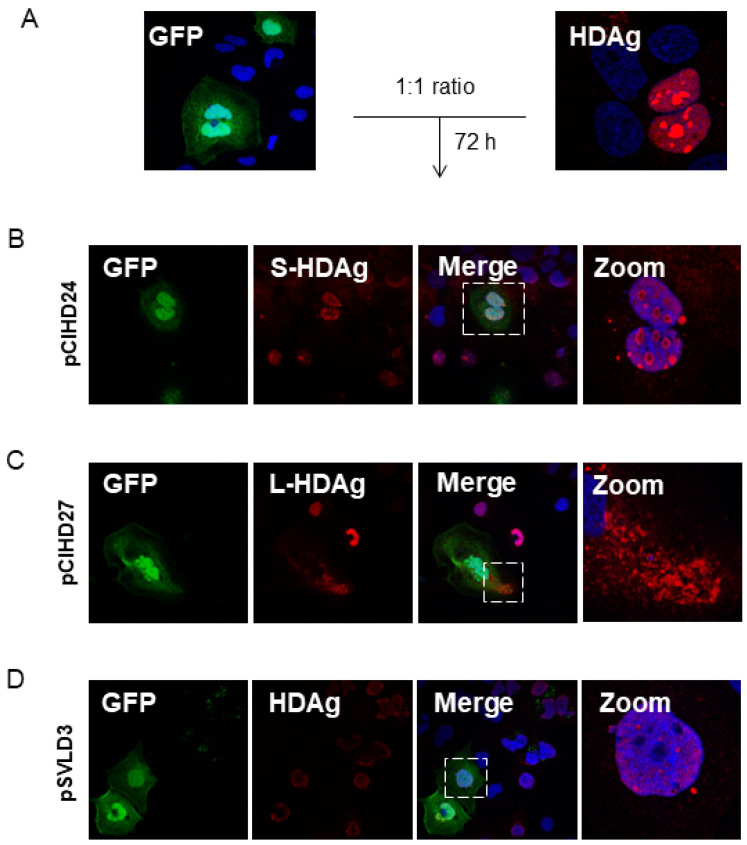
HDV components propagate between hepatocytes. (**A**) Huh7 cells were transfected with pSVLD3, pCIHD24, or pCIHD27 separately. Each pool of transfected cells was then mixed and co-cultured with cells transfected with peGFP separately for 72 h. After fixing, cells were stained with anti-HDAg antibody (red) and DAPI (blue). The results were analyzed using confocal microscopy, and a representative example was presented. (**B**) Cells transfected with pCIHD24 (S-HDAg) and co-cultured with peGFP (green)-transfected cells. (**C**) Cells transfected with pCIHD27 (L-HDAg) and co-cultured with peGFP-transfected cells. (**D**) Cells transfected with pSVLD3 (HDV RNP) and co-cultured with peGFP-transfected cells. The experiment was repeated at least three times.

To confirm that this observation is biologically significant and not due to a technical artifact, we transfected the Huh7 cells separately with either peGFP, pmRuby2, pSVLD3, pCIHD24, or pCIHD27 plasmids. We co-cultured the different transfected cells with GFP-expressing cells for 72 h and analyzed protein expression using FACS (Figure 3A) and confocal microscopy (Figure 4). Our hypothesis was that HDAg produced by cells transfected with pSVLD3, pCIHD24, or pCIHD27 plasmids would spread to GFP-positive Huh7 cells and give rise to a double-positive population (GFP+, HDAg+). GFP-transfected cells co-cultured with pmRuby2-transfected cells were used as negative control. The results depicted in Figure 3 and Figure 4 clearly demonstrate that HDAg from pSVLD3-transfected cells and pCIHD24-transfected cells can significantly propagate to the GFP-transfected cells compared with the negative control (peGFP and pmRuby2 co-cultures). However, the propagation of L-HDAg was less pronounced in this experiment when compared to S-HDAg (Figure 3A). The percentage of double-positive cells detected upon the co-culture of GFP-transfected cells with pmRuby2-transfected cells (negative control) was low, which indicates that the ability to propagate is specific to HDAg. These results demonstrate that HDAg can propagate in the absence of HBV envelope proteins and HDV virions. Interestingly, the proportion of double-positive cells was significantly higher upon pSVLD3 transfection when compared to pCIHD24 and pCIHD27 transfection. This was somehow surprising since the expression level from pCIHD24 and pCIHD27 is from a CMV promoter that leads to higher expression compared with pSVLD3 expression, as observed using western blotting on day 2 post-transfection (Figure 3B, left). In pSVLD3-transfected cells, the replication of the HDV genome occurs along with the synthesis of the HDAg isoforms, assembling into a ribonucleoprotein structure. We hypothesized from our results that this structure promotes the spreading of HDV components in a more efficient manner than S- or L-HDAg alone. Furthermore, the S-HDAg isoform was more efficient in propagating than L-HDAg, even though their expression level were similar (Figure 3A,B). The results of confocal microscopy, using the same experimental setting, also showed the apparition of double-positive cells expressing HDAg and GFP (Figure 4A–C). These observations were maintained for cells expressing the HDV RNP (Figure 4C). Interestingly, we observed that the propagation of the S-HDAg gives rise to both the nuclear and cytoplasmic localization of the protein (Figure 4A), whereas mostly cytoplasmic localization was observed for L-HDAg (Figure 4B). Of note is the propagation of HDV RNP in GFP-expressing cells, which resulted in HDAg, localizing mostly in the nucleus (Figure 4C).

### 3.2. HDAg Expression Increases Extracellular Vesicle Secretion

**Figure 5:**
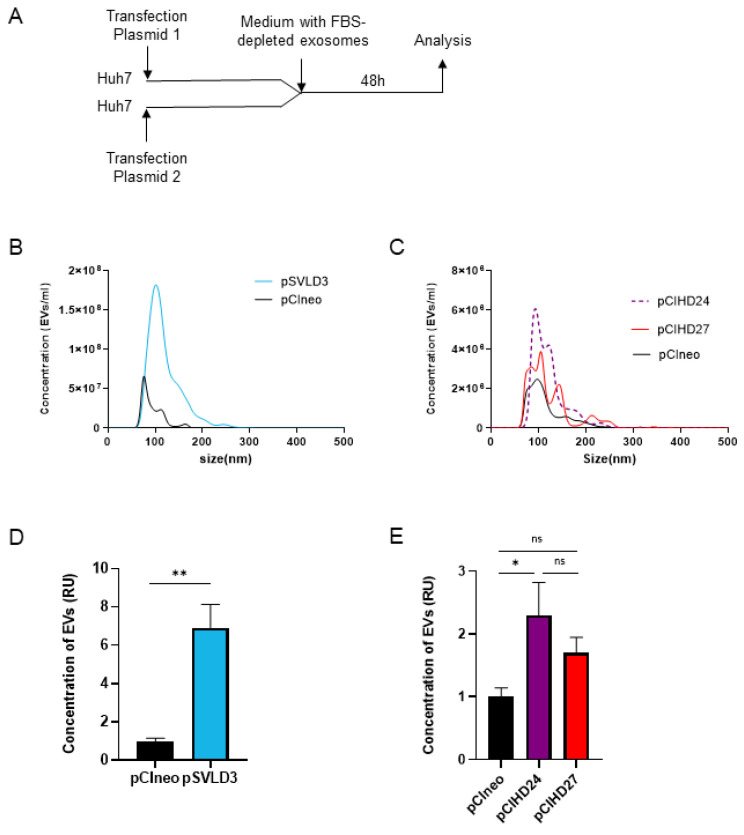
HDAg expression increases the concentration of extracellular vesicles. (**A**) Huh7 cells were transfected with pCIneo, pSVLD3, pCIHD24, or pCIHD27. Then, cells were washed, and media were replaced with a DMEM complemented with exosome-depleted FBS. After 48 h of accumulation, supernatant was collected and analyzed with NANOSIGHT NS300. (**B**) The concentration and the size distribution of extracellular vesicles (EVs) from pSVLD3- and pCIneo-transfected cells were presented. (**C**) The concentration and the size distribution of EVs from pCIHD24, pCIHD27, and pCIneo were shown. The concentration of EVs secreted from pSVLD3- (**D**) or pCIHD24/27- (**E**) transfected cells is presented as relative units (RU) normalized to the concentration of EVs secreted from pCIneo-transfected cells. The values represent the mean of three separate experiments. ns, not significant; * *p* < 0.05; ** *p* < 0.01.

The exosomes are nano-sized (50–150 nm) extracellular vesicles (EVs). To investigate the concentration and the size distribution of extracellular vesicles secreted in the supernatant of Huh7 cells upon transfection with pCIneo, pCIHD24, pCIHD27, or pSVLD3, we used the NANOSIGHT NS300 (Figure 5A). We note that when transfected with pSVLD3 (expressing HDV RNP), cells secreted significantly more EVs compared with pCIneo-transfected cells (Figure 5B and Figure 5D, respectively). In addition, the size distribution of EVs secreted from pSVLD3-transfected cells was different compared with EVs secreted from pCIneo-transfected cells: for pSVLD3, the size was 100–150 nm, while it is less than 100 nm for pCIneo (Figure 5B). This suggests that the composition of EVs from pSVLD3-transfected cells is different compared with that of EVs from pCIneo-transfected cells. The size distribution of EVs from pCIHD24-(S-HDAg) and pCIHD27-(L-HDAg) transfected cells was similar; however, only the concentration of EVs from pCIHD24-transfected cells (S-HDAg) was significantly higher than that of EVs from pCIneo-transfected cells (Figure 5C,E). Interestingly, the concentration of EVs secreted from cells transfected with pSVLD3 was higher than cells transfected with only pCIHD24 or pCIHD27 (Figure 5D,E), consistent with the previous observation from Figure 3A suggesting a higher propagation of HDV when both S- and L-HDAg are present.

### 3.3. HDV S-HDAg and L-HDAg Are Present in Exosomes

**Figure 6:**
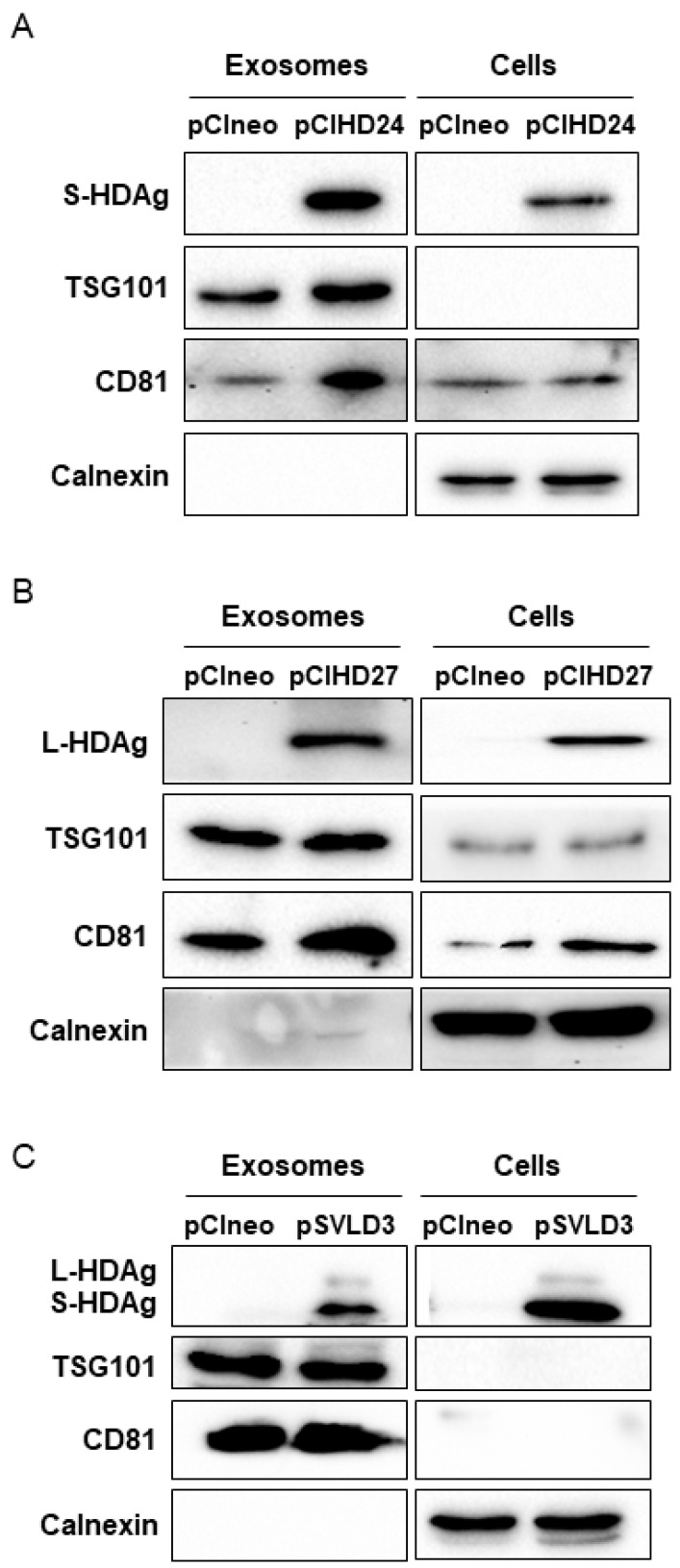
Exosomes contain HDAg. Two sets of transfections were performed; the first set was Huh7 cells transfected with pSVLD3 or pCIneo. The media was replaced with DMEM complemented with exosomes-depleted FBS 6 days post-transfection. The second set is composed of Huh7 cells transfected with pCIHD24, pCIHD27, or pCIneo. At 3 days post-transfection, media was replaced with DMEM complemented with exosomes-depleted FBS. After 48 h, cells and supernatant from both sets were harvested. Proteins extracted from exosomes and from cells transfected with pCIHD24 (**A**), pCIHD27 (**B**) or pSVLD3 (**C**) were analyzed using western blot as indicated. For each set of transfected cells, the expression of exosome markers (TSG101 and CD81) and an endoplasmic reticulum marker (calnexin) was evaluated in cellular and exosomal extracts in addition to HDAg expression. One representative of at least three independent blots is shown for each condition.

We then sought to explore the possibility that the HDAg might be present specifically in the exosomes. To this end, we purified exosomes from supernatant of Huh7 cells transfected with pCIneo, pCIHD24, pCIHD27, or pSVLD3 and cultured in exosome-free FBS media to avoid contamination with bovine exosomes. Moreover, HDV virions are not secreted from the transfected cells due to the absence of HBV envelope protein expression in our setting. Notably, the exosomes from pSVLD3-transfected cells were collected 8 days post-transfection, a time point at which the edition process of the HDV RNA has occurred, leading to the expression of both S-HDAg and L-HDAg and to the production of mature RNPs containing both HDAg isoforms. Some proteins, such as TSG101 (an ESCRT-I component) and tetraspanins (CD81, CD9), are enriched in exosomes and are, therefore, commonly used as markers to characterize exosomes [48,49,50]. Samples were analyzed using western blotting, and TSG101 and CD81 were used to identify exosomes. As a control for purification, we monitored the presence of the ER-resident protein calnexin [50,51], which is expected to be absent from exosome preparations. Equal protein amounts of extracts prepared from cells or exosomes were subjected to western blotting analysis (Figure 6). As expected, calnexin was detected in cellular extracts but was absent from the exosome preparations (Figure 6A–C). These results demonstrate that the exosome preparation was not contaminated with material derived from other cellular compartments. Importantly, we detected a clear enrichment of exosomal proteins (TSG101 and CD81) in our preparations (Figure 6A–C), supporting the postulate that the purified vesicles are indeed exosomes [51]. Exosomes derived from pCIHD24- and pCIHD27-transfected cells contain S-HDAg and L-HDAg, respectively (Figure 6A,B), demonstrating that incorporation of the isoforms of HDAg in the exosomes does not require the HDV viral genome. Additionally, the exosomes isolated from pSVLD3-transfected cells contain both HDAg isoforms (Figure 6C). These results confirm the presence of HDAg proteins in exosomes.

### 3.4. Exosomes Harbour HDV RNA

**Figure 7:**
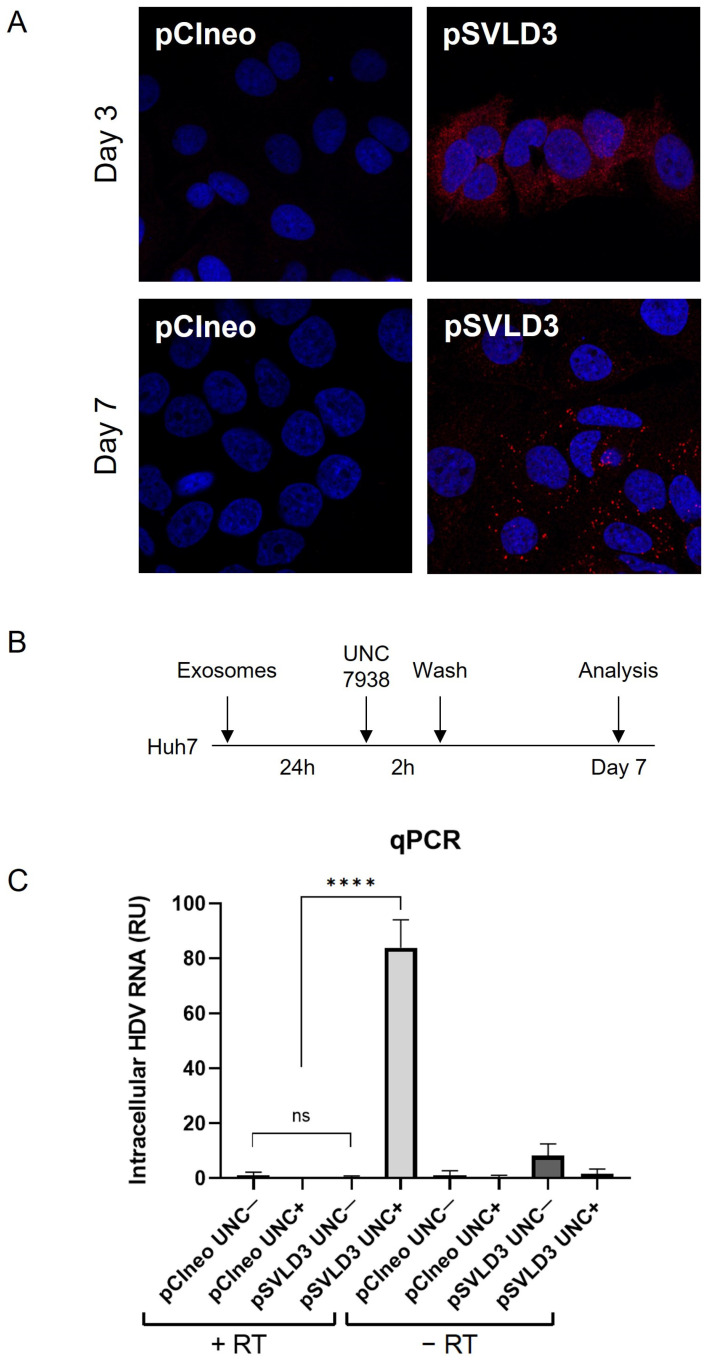
Exosomes deliver HDV components to targeted naïve cells. Naïve Huh7 cells were cultured with 100,000 exosomes/cell derived from pSVLD3-transfected cells or pCIneo-transfected cells for 3 days or 7 days. (**A**) Cells were fixed and stained with anti-HDAg antibody (red) and DAPI (blue). (**B**) Huh7 were cultured with 100,000 exosomes/cell (originating from pSVLD3- or pCIneo-transfected cells). After 24 h, UNC7938 (15 µM) was added for 2 h and then washed. Seven days later, cells were lysed. (**C**) Intracellular RNAs harvested were either submitted to RT or not (control) and analyzed using qPCR. ns, not significant; **** *p* < 0.0001.

Following the detection of HDAg within exosomes and knowing that these vesicles mediate cell-to-cell communication [3] and receptor-independent transmission of viruses [13], we sought to evaluate the ability of the exosomes derived from pSVLD3-transfected cells to transfer their content (HDAg and/or viral RNA) to naïve hepatocytes cells. To this end, we exposed naïve Huh7 cells to purified exosomes from pSVLD3- or pCIneo-transfected cells. Cells were then stained using an anti-HDAg antibody and analyzed using confocal fluorescence microscopy at days 3 and 7 post-exposure (Figure 7A). On day 3, the staining appeared to localize in a cytoplasmic subcellular compartment (likely endosomes), while on day 7, more intense puncta could be observed in addition to the structures observed on day 3 (Figure 7A). Although it cannot be completely ruled out, neosynthesized HDAg on day 7 should result in stronger nuclear staining [52]. At this stage, it seems more likely that the HDV components (HDAg and genome) remain trapped in endosomal and lysosomal structures. It is known that, in vitro, nucleic acids cannot be released in the cytoplasm and remain trapped in late endosomes [41,53]. Interestingly, the UNC7938 compound has been shown to release oligonucleotides from endosomes in vitro [41,42,53]. Based on the observation of punctuated staining of HDAg in cells, likely resulting from endosomal/lysosomal sequestration, we sought to investigate the ability of UNC7938 to release HDV components and to initiate the novo HDV RNA replication (Figure 7B). On day 7 post-inoculation, cells were analyzed for their HDV RNA content using RT-qPCR. The results show a significant increase in signal intensity in exosome-treated cells in the presence as compared to the absence of UNC7938 treatment (Figure 7C). Importantly, total RNA was also subjected to a mock reverse transcription prior to qPCR to ascertain the absence of plasmid contamination. The increase in total cellular HDV-RNA upon treatment with UNC7938 strongly suggests that the released viral RNA was able to replicate. Altogether, these findings demonstrate that exosomes contain HDV RNA and further suggest the ability of the released viral RNA (upon UNC7938 treatment) to replicate in the target cell.

## 4. Discussion

Infection by HDV usually leads to the aggravation of liver dysfunction and higher risks of severe liver disease [25,54] as compared to HBV mono-infected patients. Originally known as an HBV satellite virus, a recent publication suggested that HDV can exploit other enveloped viruses unrelated to HBV to complete its life cycle and propagate to naïve cells [40]. Moreover, the receptor-independent transmission of viruses through exosomes has been demonstrated for many viruses, including HIV, HCV, HAV, and HBV [13,19,21,22,55,56,57]. However, the transmission of HDV via exosomes secreted from hepatocytes has never been reported. Recently, it was demonstrated that extracellular vesicles from HDV-infected cells harbour viral HDV RNA and induce a proinflammatory cytokine response in human peripheral blood mononuclear cells and macrophages [23]. Such studies are important for gaining a better understanding of how viruses hijack the membrane trafficking of the host cell machinery to spread to neighbouring and distant cells and to modulate the host immune response.

Our first observations have shown that HDAg expressed from Huh7-transfected cells can be detected in the surrounding non-transfected cells. Accordingly, we have investigated and verified whether the HDAg was transferred from transfected to non-transfected cells. The initial observations were confirmed in a model of co-culture of peGFP-transfected cells and HDAg-transfected cells. Interestingly, in Figure 1 and Figure 2, as well as in Figure 4A,B, the localization of L-HDAg in the surrounding cells and double-positive cells is mostly cytoplasmic. Furthermore, the localization of S-HDAg in the surrounding cells and double-positive cells can be either nuclear or cytoplasmic. As well, the percentage of double-positive cells in cells expressing S-HDAg was significantly higher compared to cells expressing L-HDAg only. These results together suggest that the propagation of S-HDAg can be either caused by cell-to-cell transmission through direct cellular contact or by transmission between distant cells mediated by extracellular vesicles. Conversely, L-HDAg propagation is mostly due to cell-to-cell transmission by direct cellular contact. However, we cannot ignore the fact that L-HDAg can still propagate through its incorporation into exosomes, as demonstrated by the confocal microscopy and the western blotting results.

It has been demonstrated that different hepatitis viruses exploit the exosomes either to be engulfed, like HEV [20] and HAV [58], or to transmit the infection to other hepatocytes, like HBV and HCV [21,22,59]. Therefore, we hypothesized that exosomes might be involved in a virion-free propagation of HDV components. In this study, we demonstrated that the expression of RNP significantly increases the concentration of secreted extracellular vesicles. Most of these vesicles have a size that ranges approximately between 100 nm and 150 nm, the typical size of exosomes (50–150 nm) [3], suggesting that the extracellular vesicles quantified might be mostly exosomes. In addition, we showed that the size distribution of extracellular vesicles from cells expressing RNP was different compared to extracellular vesicles derived from pCIneo-transfected cells, suggesting a modification in the composition of extracellular vesicles upon expression of the HDV RNP. This possibility should be investigated in future work. Interestingly, recent publications have demonstrated the ability of the HBx protein of HBV to increase the secretion of extracellular vesicles, including exosomes [60], and that the composition of exosomes can be modified in infected HBV cells compared with uninfected cells [61], corroborant with our observation with HDV.

Further, our molecular characterization of secreted EVs using western blotting shows clearly that these EVs are enriched with exosomal protein markers (TSG101 and CD81), confirming that these EVs are exosomes. Moreover, they do not express ER-resident protein calnexin, thereby confirming that the exosome preparation was not contaminated with material derived from other cellular compartments. Isolated exosomes from pSVLD3 transfected Huh7 supernatant contains HDAg (Figure 6B–D), as well as HDV RNA (Figure 7C). Therefore, we speculate that these components could be internalized into exosomes as a structured HDV RNP. This hypothesis is reinforced by the enhanced propagation of HDAg observed upon transfection with pSVLD3 as compared to HDAg (p24 or p27 only) expressing plasmids (Figure 3B). Indeed, while the HDV promoter in pSVLD3 was weaker and produced less HDAg (Figure 3B) than pCIHD24 or pCIHD27, which harbour a CMV promoter, more HDAg was observed in the neighbouring cells transfected with pSVLD3 (Figure 3A). These observations could also be explained by the fact that the pSVLD3 promoter launches a rolling circle replication of the RNA, increasing the production of HDAg over time. This amplification process was not present on pCIHD24 and pCIHD27 plasmids. Also, when cells transfected with pSVLD3 were co-cultured with peGFP-transfected cells, we observed the presence of double-positive cells with mostly nuclear localization of HDAg (Figure 4D). However, when Huh7 cells were cultured with purified exosomes from pSVLD3-transfected cells, we observed a cytoplasmic localization of HDAg (Figure 7A). These results suggest that there may be two different mechanisms of RNP transmission between cells: cell-to-cell by direct contact between neighbouring cells, which is reflected by nuclear localization, or mediated by extracellular vesicles, including exosomes. The cytoplasmic localization of HDAg seems more likely due to the entrapment of the HDV components (HDAg and genome) in endosomal and lysosomal structures. Our study shows that endosomal release, using UNC7938, significantly increases the quantity of the viral genome detected in cells exposed to exosomes. It is known that in vitro, it is challenging for the oligonucleotides to escape the endosomal pathway and reach their targets [62]. Likewise, in the absence of an endosome-disrupting agent, HDV components (including the genome) remain trapped in endosomal structures. However, when treated with UNC7938, cells exhibit a significantly increased level of HDV RNA, strongly suggesting the occurrence of de novo viral genome replication.

Our results suggest that exosomes contribute to the propagation of HDV infection. However, a deeper analysis of the virus-free propagation of the HDV genome and additional analysis of the rolling-circle replication re-initialization in naïve cells is warranted. The propagation of HDV by the endosomal pathway could be to the advantage of the virus through its escape from the immune response or to adversely enhance the innate immune response and trigger the activation of immune cells. It has been demonstrated for HBV that exosomes secreted from hepatocytes deliver viral proteins to recipient cells, like monocytes, macrophages, and NK cells, so as to regulate the immune response [21,61,63]. Furthermore, it has been recently shown that extracellular vesicles derived from HDV-infected cells induce a proinflammatory cytokine response in macrophages and human peripheral blood mononuclear cells [23]. We assume that exosomes containing HDV components may modulate the immune response and thereby explain the observation; in contrast to mono-infection with HBV, HBV–HDV co-infection leads to a high level of activation of the innate immune system [64]. Exosome-containing HDV components may also explain the observation that HDV can persist in cells for weeks in the absence of HBV and for months after liver transplantation [65]. Recently, it has been shown that exosomes derived from HCV-infected hepatocytes are internalized within the hepatic stellate cells (HSCs) and increase the expression of profibrotic markers by the activated HSCs. Therefore, the HCV exosomes may be implicated in the induction of liver fibrosis [66]. It is also known that the presence of HDV in the liver accelerates the pathogenesis of liver diseases, including fibrosis [67]. The causes of this rapid progression are not well known, but the strong induction of exosome secretion by HDV replicating cells may contribute to the pathogenesis and fibrosis caused by HDV, as observed with HCV [66]. This possibility should be evaluated in future work.

In summary, we have shown that HDV viral components are present in exosomes and are able to enter naïve hepatocytes. These exosomes mediate the transfer of HDAg and the viral RNA between hepatocytes in vitro, independently of HBV. Endosomal disruption experiments further suggest that cells exposed to HDV-containing exosomes can initiate de novo replication. Therefore, exosomes are likely involved in the propagation of HDV to other hepatocytes in the absence of HBV, contributing to its persistence in the liver. Therefore, additional studies will be required to analyze the contribution of exosomes in the maintenance of HDV in the liver of patients during clinical treatment targeting HBV replication or HBsAg production.

## Data Availability

Data are available upon request from the corresponding author.

## Abbreviations

HBV: hepatitis B virus; HDV, hepatitis delta virus; HCV, hepatitis C virus; HAV, hepatitis A virus; HEV, hepatitis E virus, HIV human immunodeficiency virus; HSV-1, herpes simplex virus-1; HHV-6, human herpes virus 6; NTCP, sodium taurocholate co-transporting polypeptide; HBsAg, hepatitis B surface antigen; L-HDAg, hepatitis delta large antigen; S-HDAg, hepatitis delta small antigen; MVB, multivesicular bodies; ADAR1; adenosine deaminase RNA specific 1; NES, nuclear export signal.
